# *Stomoxys calcitrans*, mechanical vector of virulent *Besnoitia besnoiti* from chronically infected cattle to susceptible rabbit

**DOI:** 10.1111/mve.12356

**Published:** 2019-01-21

**Authors:** S. Sharif, P. Jacquiet, F. Prevot, C. Grisez, I. Raymond‐Letron, M. O. Semin, A. Geffré, C. Trumel, M. Franc, É. Bouhsira, E. Liénard

**Affiliations:** ^1^ Département Élevage et Produits–Santé Publique Vétérinaire, Laboratoire de Parasitologie et Maladies Parasitaires, École Nationale Vétérinaire de Toulouse (ENVT) Université de Toulouse Toulouse France; ^2^ Département Santé Animale, Interactions Hôtes–Agents Pathogènes (IHAP), Institut National de la Recherche Agronomique (INRA), ENVT Université de Toulouse Toulouse France; ^3^ Département Santé Biologiques et Fonctionnelles, Laboratoire d'HistoPathologie Expérimentale et Comparée (LabHPEC), ENVT Université de Toulouse Toulouse France; ^4^ STROMALab, Université de Toulouse, CNRS ERL5311, EFS, ENVT, Institut National de la Santé et de la Recherche Médicale (INSERM) U1031 Université de Toulouse Toulouse France; ^5^ Département Sciences Cliniques Des Animaux De Compagnie, Équipe de Biologie Médicale‐Histologie, Centre Régional d'Exploration Fonctionnelle et de Ressources Expérimentales, INSERM, ENVT Université de Toulouse Toulouse France

**Keywords:** *Besnoitia besnoiti*, bradyzoites, immunoblot, qPCR, rabbit, stable fly, IFAT

## Abstract

Cattle besnoitiosis caused by *Besnoitia besnoiti* (Eucoccidiorida: Sarcocystidae) is a re‐emerging disease in Europe. Its mechanical transmission by biting flies has not been investigated since the 1960s. The aim of this study was to re‐examine the ability of *Stomoxys calcitrans* (Diptera: Muscidae) to transmit virulent *B. besnoiti* bradyzoites from chronically infected cows to susceptible rabbits. Three batches of 300 stable flies were allowed to take an interrupted bloodmeal on chronically infected cows, followed by an immediate bloodmeal on three rabbits (Group B). A control group of rabbits and a group exposed to the bites of non‐infected *S. calcitrans* were included in the study. Blood quantitative polymerase chain reaction (qPCR) analyses, and clinical, serological and haematological surveys were performed in the three groups over 152 days until the rabbits were killed. Quantitative PCR analyses and histological examinations were performed in 24 tissue samples per rabbit. Only one rabbit in Group B exhibited clinical signs of the acute phase of besnoitiosis (hyperthermia, weight loss, regenerative anaemia and transient positive qPCR in blood) and was seroconverted. Parasite DNA was detected in four tissue samples from this rabbit, but no cysts were observed on histological examination. These findings indicate that *S. calcitrans* may act as a mechanical vector of *B. besnoiti* more efficiently than was previously considered.

## Graphical abstract

This is the first reproduction of the mechanical transmission of *Besnoitia besnoiti* by stable flies in a heterologous model.
A batch of 300 stable flies was sufficient to transmit virulent *B. besnoiti* bradyzoites from chronically infected cattle to rabbit.
Transmission was effective: one infected rabbit exhibited clinical signs of acute besnoitiosis and seroconversion.

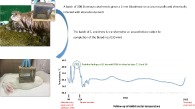

## Introduction

Cattle besnoitiosis caused by the cyst‐forming parasite *Besnoitia besnoiti* (Henry, 1913) has garnered more attention in the two past decades since recent outbreaks in various European countries led the European Food Safety Authority (EFSA) to categorize this disease as re‐emerging (EFSA, 2010). During the early and acute stages of the disease, a rapid proliferation of tachyzoites occurs within the endothelial cells of blood vessels and causes vascular lesions (Pols, 1960; Basson *et al*., 1970). The main clinical signs associated with parasite multiplication are fever, nasal and ocular discharges and, later, subcutaneous oedema (anasarca stage). Sporadic (but economically important) abortion, male (reversible or not) sterility and death may occur (Pols, 1960; Bigalke, 1968; Jacquiet *et al*., 2010). The final and chronic stage of the infection in cattle includes scleroderma, hyperkeratosis, hair loss, and the folding, hardening and thickening of the skin with an oozing sero‐sanguineous exudate (Bigalke & Prozesky, 2004). Characteristic sub‐spherical, pinhead‐sized, thick‐walled cysts arise around 11 days post‐infection in various organs and tissues, although mainly in the skin and scleral conjunctiva (Bigalke, 1968), and may contain up to approximately 200 000 bradyzoites (Bigalke, 1981).

The spreading of the infection within and between cattle herds remains poorly understood (Olias *et al*., 2011). Bigalke (1968) investigated one of the possible routes of contamination, collecting evidence that blood‐sucking arthropods are able to mechanically transmit *B. besnoiti* from chronically infected cattle to susceptible animals. These results suggested that tabanids are likely to be dramatically more efficient vectors than the worldwide common pest *Stomoxys calcitrans* Linnaeus, 1758 (Bigalke, 1968). However, some seroconversions were observed in housed dairy cattle at the end of winter (i.e. outside the horsefly activity period) at a time concomitant with high indoor activity in *S. calcitrans* (Liénard *et al*., 2011). This supports the suggestion of vector competence for the transmission of *B. besnoiti* in stable flies. The rabbit, *Oryctolagus cuniculus* (Lagomorpha: Leporidae), has been found to be a susceptible host of *B. besnoiti* (Pols, 1960; Bigalke, 1968; Basson *et al*., 1970; Liénard *et al*., 2015). Therefore, the use of the rabbit as an experimental animal in place of cattle provides an alternative cheap and easy model. The purpose of this study was to reassess the vector competence of the stable fly to transmit virulent *B. besnoiti* bradyzoites from chronically infected cattle to susceptible rabbits under laboratory conditions.

## Materials and methods

### Rearing of *S. calcitrans* colony

A laboratory colony of *S. calcitrans* has been reared under laboratory conditions at the École Nationale Vétérinaire de Toulouse (ENVT) according to Salem *et al*. (2012) since 2009. A total of 2400 newly emerged stable fly males and females (sex ratio: 1 : 1) with a mean ± standard deviation age of 4 ± 2 days were used in this study.

### Source of *B. besnoiti* bradyzoites

Two Blonde d'Aquitaine cows (cow 1 and cow 2) naturally and chronically infected with *B. besnoiti* and in the scleroderma stage of disease were referred to the ENVT large animal hospital for necropsy. These animals had been provided by commercial herds in the French departments of Aude and Cantal and were used as sources of *B. besnoiti* bradyzoites. *Besnoitia besnoiti* infection was confirmed by serology using in‐house western blot (WB) analysis and by real‐time quantitative polymerase chain reaction (qPCR) performed on neck skin biopsy material.

### Rabbits as receiver hosts

Seven female, 12‐week‐old New Zealand White rabbits ranging in weight from 2.3 kg to 2.8 kg were obtained from the experimental unit Pôle Expérimental Cunicole de Toulouse of the Institut National de la Recherche Agronomique (Castanet‐Tolosan, Toulouse). Rabbits were individually housed in cages in a room with controlled relative humidity (65–70%) and temperature (20–22 °C). The animals were fed with vegetable pellets daily. Fresh tap water was available ad libitum. Animals were acclimatized for 2 weeks prior to the experiments and were handled daily. These experiments were approved by the ethics committee of the ENVT (Science et Santé Animale; agreement no. 115) under the reference APAFIS#7628‐2016111717356142v4. Before the experiments, all rabbits were confirmed as negative for anti‐*B. besnoiti*‐specific antibodies by in‐house WB analysis and by indirect fluorescent antibody test (IFAT) and as negative for *B. besnoiti* DNA in blood by qPCR.

### Experiment design

Stable flies were fed with cattle blood and honey at 48 h and 24 h before the experiments. The right side of the neck of each cow was shaved and disinfected with iodopovidone solution (Vetedine Solution Externe 120 mL; Laboratoire Vétoquinol SA, Lure, France) and alcohol 70% at 3 h prior to the experiment.

#### Assessment of engorgement rate of *S. calcitrans* on cows

Three batches of 300 stable flies were isolated in three mesh cages (15 × 15 × 15 cm). Each batch was exposed to the neck of one cow over 5 min; two batches were placed on cow 1 and one batch was placed on cow 2. Flies were killed by freezing to − 20 °C immediately after the bloodmeal. In each batch, the engorgement rate was assessed by needle abdominal dissection. Proboscises were then removed and pooled by batch in a grinding tube containing 1.4 mL of phosphate‐buffered saline (PBS) (Bio‐Rad France SA, Marnes‐la‐Coquette, France). Collected abdominal contents were pooled per batch in sterile 4‐mL tubes containing EDTA (Terumo Europe NV, Leuven, Belgium) and 2 mL of PBS. Quantitative PCR for *B. besnoiti* DNA was performed on the abdominal contents and mouthparts of each batch of flies.

#### Experimental infection

The rabbits were divided randomly into the following three groups. In ‘group bradyzoites’ (Group B), three rabbits (B1, B2, B3) received bites from stable flies that had fed previously on cows chronically infected with *B. besnoiti*. In ‘group *Stomoxys*’ (Group S), two rabbits (S1, S2) were bitten only by *S. calcitrans* that were free of *B. besnoiti* infection. The control group (Group C) included two rabbits (C1, C2). On the day of the experiment, all rabbits were anaesthetized by i.m. injection of ketamine hydrochloride (15 mg/kg, Imalgene 1000®; Merial SA, Lyon, France) and medetomidine hydrochloride (0.25 mg/kg, Domitor®; Laboratoire Vétoquinol SA). A 15 × 15 cm patch was shaved on the right flank of each rabbit.

Experiment 1 involved the transfer of *B. besnoiti* bradyzoites from chronically infected cows to rabbits (Group B) using stable flies. In this experiment, three batches of 300 stable flies were allowed to take bloodmeals from two chronically infected cows for 5 min as an interrupted bloodmeal (Liénard *et al*., 2013); batches 1 and 2 were placed on cow 1, and batch 3 was placed on cow 2. The batches of flies were contained in the same cages as described for the assessment of the engorgement rate of *S. calcitrans* on cows. Batches 1, 2 and 3 were immediately transferred and allowed to complete their bloodmeals on rabbits B1, B2 and B3, respectively, for 30 min.

To avoid the passive transfer of the parasite by mesh cages, different faces of the same cage were presented to the donor cattle and rabbits. At the end of the second bloodmeal, the stable flies were killed by freezing to − 20 °C.

Experiment 2 examined the effects of *B. besnoiti*‐free stable fly bites on rabbits (Group S). Two batches of 300 stable flies were prepared as described in Experiment 1 and fed for 5 min on 960 µL of *B. besnoiti*‐free cattle blood, divided into 48 drops of 20 µL, distributed on 68‐well slides (Gerhard Menzel GmbH, Braunschweig, Germany) and heated at 38 °C. These batches were immediately transferred to rabbits S1 and S2 and allowed to complete their bloodmeals for 30 min. Then, they were killed by freezing to − 20 °C.

### Clinical examination

All rabbits were clinically monitored. Rectal temperature and bodyweight were recorded at days 4 and 2 before exposure to stable fly bites, every day from day 0 to day 28 and weekly from day 22 to day 152 post‐exposure (p.e.) to *S. calcitrans*. All rabbits were killed on day 152 by injection into the marginal ear vein of 0.12 mL/kg bodyweight of T‐61® containing embutramide 200 mg/mL, mebezone iodure 26.92 mg/mL and tetracaine chloridrate 4.39 mg/mL (Intervet SA, Beaucouzé, France).

### Laboratory procedures

### *Serological and complete blood count examinations*

Blood was taken from the cephalic vein by venipuncture (Surflo® Winged Infusion Set; Terumo Europe NV) on days − 2, 2, 7, 9, 14, 16, 21, 23, 28, 35, 42, 49, 56, 63, 77, 91, 105, 119, 133 and 147. Blood was collected in 4‐mL tubes containing EDTA (Terumo Europe NV) to assess the parasite load by qPCR and in 1‐mL tubes containing EDTA (Greiner Bio‐One GmbH, Kermsmünster, Austria) for haematological analysis. Serum samples collected in 3.5‐mL tubes containing silicone (Terumo Europe NV) were tested for *B. besnoiti* antibodies by in‐house WB using total *B. besnoiti* tachyzoite antigens and IFAT.

The in‐house WB and IFAT procedures were performed as previously detailed by Liénard *et al*. (2015) for rabbit blood samples. Western blotting was also used for cattle blood samples. For both immunological tests in rabbits, serum from one previously bradyzoite‐infected rabbit was used as a positive control (Liénard *et al*., 2015). Negative controls were obtained from uninfected control rabbits. For cattle blood samples, a positive control sample was obtained from a chronically *B. besnoiti*‐infected cow and a negative control was sourced from an uninfected cow. In WB, a serum sample was considered positive when at least four of 10 bands of tachyzoite antigens (45, 40, 37, 34, 30, 27, 22, 17, 16 and 15 kDa) were recognized according to Schares *et al*. (2010). In IFATs, the 1 : 200 dilution was considered the positive cut‐off (Shkap *et al*., 2002; Lenfant *et al*., 2014) with unbroken, peripheral bright fluorescence of the tachyzoite membrane. For haematological analyses, the labelled tubes were mixed gently and stored at 4 °C until analysis within 3 h of blood collection. Haematological analyses were performed with an automated analyser (XT‐2000iV; Sysmex Corp., Kobe, Japan) using the settings for rabbit blood (XT‐iV series software Version 10; Sysmex Corp.).

Rabbit necropsy and qPCR on rabbit blood and tissues and on mouthparts and abdominal contents of stable flies

At day 152, all rabbits were submitted to necropsy. For each animal, 24 skin and tissue samples were collected for PCR, histology and immunohistochemistry analyses. Skin samples of 4 cm^2^ were taken from 10 sites, including the right fore and hind limbs, right inner thigh, backline, right flank (i.e. stable fly bite site), umbilicus area, udder, right neck, right shoulder and right eyelid. Fourteen samples of tissues and organs (1 cm^3^) were taken from the pancreas, liver, spleen, right kidney, gallbladder, diaphragm, heart, lung, nasal and tracheal mucosa, right eye, right ovary, vulvar and vaginal mucosa. Each sample weight was homogenized to 500 mg for qPCR.

Detection of *B. besnoiti* DNA was performed with qPCR of blood (1 mL), skin, tissue and organ samples of rabbits, mouthparts and abdominal contents of *S. calcitrans*, and cattle neck skin samples (after the necropsies of cows 1 and 2). The mouthparts of stable flies were ground with the TeSeE™ Purification Kit (BioRad France SA) according to the manufacturer's recommendation. Total genomic DNA was extracted with the QIAmp® DNA Mini Kit (Qiagen SAS, Courtaboeuf, France). *Besnoitia besnoiti* internal transcribed spacer 1 (ITS‐1) amplification was performed with the commercial PCR kit AdiaVet™ Besnoitia (Bio‐X Diagnostics SA, Jemelle, Belgium) and with the Stratagene MX3005P thermal cycler (Agilent Technologies, Inc., La Jolla, CA, U.S.A.). Positive and negative template controls were included in all experiments. Results were computed using MxPro QPCR Version 4.10 (Agilent Technologies, Inc.). Threshold cycle (Ct) and baseline values were automatically determined by the software and verified by visual examination of the threshold line in amplification plots. A Ct value of ≥ 40 corresponded to any parasite DNA detection.

Assessments of parasite equivalent (par. eq.) numbers in rabbit samples (tissues and blood), mouthparts and abdominal contents of stable flies and in cattle blood samples were performed under the conditions described in Sharif *et al*. (2017). The obtained Ct values were transformed into the number of parasite equivalents according to the following equation of the linear regression of the standard curve: $\mathrm{par}.\mathrm{eq}.=10^{\left(\frac{\mathrm{ct}-40.405}{-1.534}\right)}$ estimated using Microsoft™ Excel 2013 (Microsoft Corp., Redmond, WA, U.S.A.).

Histology and iImmunohistochemistry of rabbit skin and tissue samples

After qPCR results had been obtained, 10% buffered formalin was used to fix mirror samples in order to evaluate the presence of *B. besnoiti* cysts in rabbits. These samples were dehydrated, embedded in paraffin wax and 4‐µm sections were stained with haematoxylin and eosin. Full‐thickness serial sections of the paraffin blocks were obtained in PCR‐positive mirror samples to assess the development of bradyzoite cysts or inflammatory conditions. Additionally, immunohistochemistry was performed in these samples using a peroxidase‐based staining method with polyclonal antibodies provided by a cow naturally infected with *B. besnoiti* (serum characterized by WB and IFAT). These polyclonal antibodies were used at a dilution rate of 1 : 25 over 1 h. Slides were then incubated for 25 min with antibovine peroxidase solution [dilution 1 : 1000, antibovine immunoglobulin G (IgG) (whole molecule); peroxidase antibody produced in rabbit (Sigma‐Aldrich Chemie Sarl, Saint‐Quentin Fallavier, France)]. Diaminobenzidine was used as chromogen (DAB+; Dako France SAS, Les Ulis Cedex, France) to reveal peroxidase activity. Slides were counterstained with Harris haematoxylin.

## Results

The percentages of engorgement in batches 1 and 2 on cow 1 were very low (Table 1). No *B. besnoiti* DNA was recovered in the mouthparts or abdominal contents of the stable flies. Batches on cow 2 had a higher engorgement rate and parasite DNA was found in mouthparts and abdominal contents (Table 1). However, the parasite equivalent burden was low (Table 1).

**Table 1:** Engorgement rate and parasite burden in stable flies following 5 min of blood feeding on cows chronically infected with *Besnoitia besnoiti*.

			Number of parasite equivalents	
Control batch	Source of bradyzoites	Engorgement rate (95% CI)	In mouthparts	In abdominal content
1	Cow 1	3.33% (1.3–5.4%)	No DNA detected	No DNA detected
2	Cow 1	16.67% (12.5–20.9%)	No DNA detected	No DNA detected
3	Cow 2	70.5% (65.3–75.7%)	9	41

CI, confidence interval.

#### Clinical follow‐up

**Figure 1:**
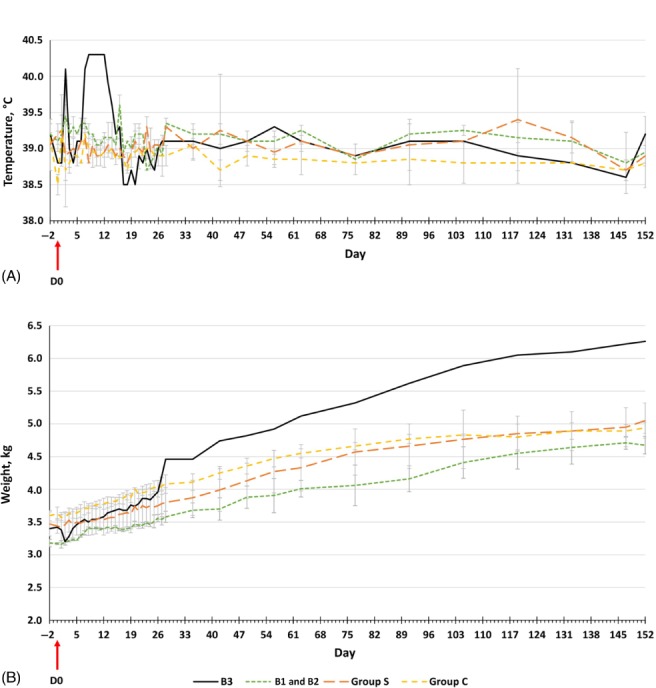
Variations in (A) mean ± standard deviation (SD) rabbit rectal temperature and (B) mean ± SD rabbit weight from day 2 to day 152. Seropositive rabbit B3 results are presented apart from those for Group B. Group B: rabbits exposed at day 0 to bites of 300 stables flies immediately transferred after an interrupted bloodmeal on chronically infected cows; Group S: two rabbits exposed only to 300 laboratory‐reared *S. calcitrans*; Group C: two rabbits anaesthetized on the day of the experiment. [Colour figure can be viewed at http://wileyonlinelibrary.com].

No clinical abnormalities were reported in rabbits within the control group or Group S, or in the two rabbits B1 and B2 infected from cow 1. No increase in rectal temperature or weight variation was found at any point during the 152‐day experiment, except in rabbit B3, which exhibited a plateau in rectal temperature for 6 days (from day 7 to day 12) of 40.3 °C over the 5 days (Fig. 1A) and at day 2 lost 180 g (Fig. 1B). No photophobia, oedema or nasal and ocular discharges were observed in any rabbit.

#### Serological, haematological and qPCR blood analyses

Positive seroconversion determined by WB and IFAT was observed in rabbit B3 only from 14 days p.e. onwards. This rabbit became seropositive with antibody titres in IFAT of ≥ 1 : 800.

**Figure 2:**
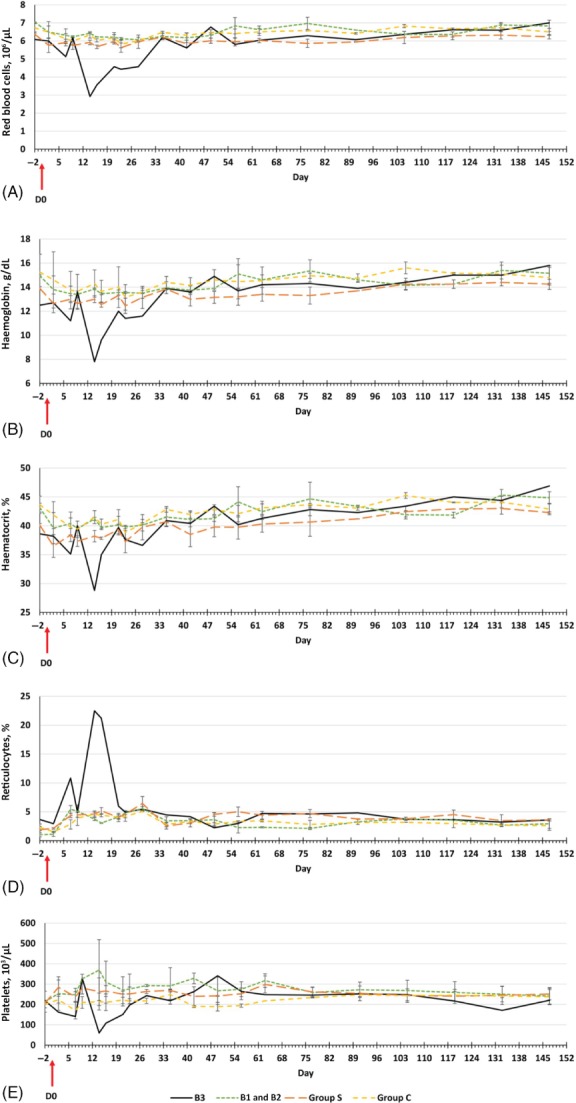
Mean ± standard deviation haematological profile variations in rabbits from day − 2 to day 147. Results in rabbit B3 are shown separately from those for Group B. (A) Red blood cell counts. (B) Haemoglobin levels. (C) Haematocrit levels. (D) Reticulocyte levels. (E) Coagulogram measured by platelet counts. [Colour figure can be viewed at http://wileyonlinelibrary.com].

**Figure 3:**
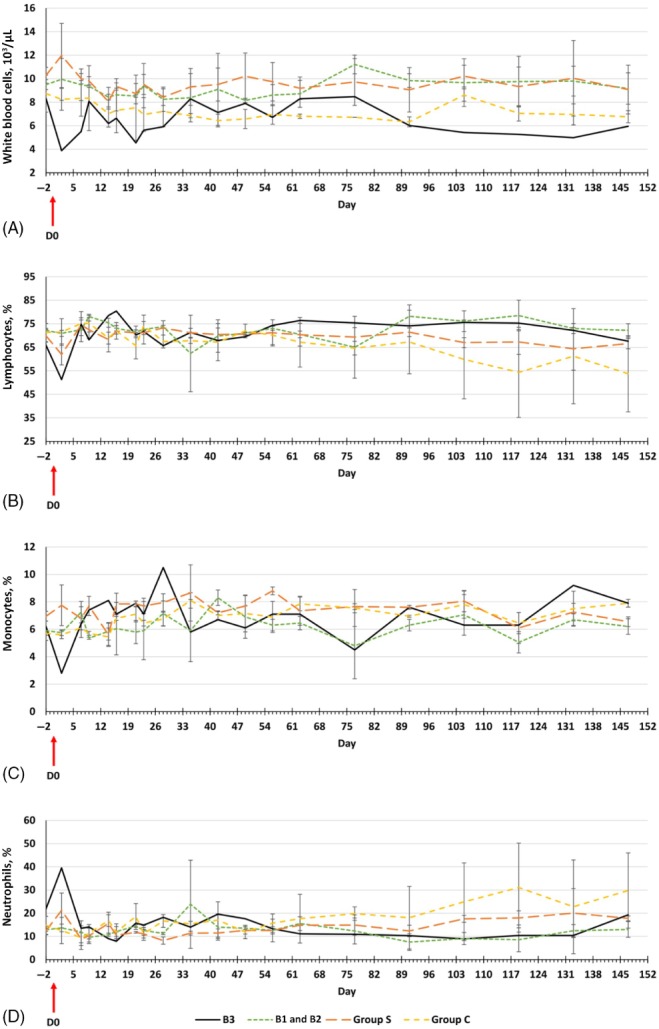
Mean ± standard deviation leukogram profile variations in rabbits from day − 2 to day 147. Results in rabbit B3 are shown separately from those for Group B. (A) White blood cell counts. (B) Lymphocyte counts. (C) Monocyte counts. (D) Neutrophil counts. [Colour figure can be viewed at http://wileyonlinelibrary.com].

Haematological variations were observed in rabbit B3 only (Figs 2 and 3): the red blood cell (RBC) count decreased at 14 days p.e. (Fig. 2A), with transient and marked anaemia, as demonstrated by low haemoglobin (Hb) and haematocrit (Ht) values (Fig. 2B, C). These increased to normal values after day 35 (Fig. 2A–C). The level of reticulocytes in rabbit B3 peaked at day 14 but fell to within the other rabbits' ranges at 23 days p.e. until the end of the study (Fig. 2D). Transient and patent thrombocytopoenia was observed from 14 days to 23 days p.e. (Fig. 2E).

A reduced white blood cell (WBC) count was observed at 2 days p.e. in rabbit B3 (Fig. 3A). The WBC count increased at 9 days p.e., fluctuated within the mean values of WBC counts in the other rabbits from 28 days to 91 days p.e. (Fig. 3A) and thereafter remained slightly lower than levels in the other rabbits. The decreased WBC count at 2 days p.e. consisted of reductions in lymphocytes and monocytes (Fig. 3B, C). A decline in neutrophils was found at 7 days p.e. (Fig. 3D). Subsequently, all of these values fluctuated within the ranges recorded in all other rabbits until the rabbits were killed (Fig. 3B–D).

*Besnoitia besnoiti* DNA was detected in rabbit B3 blood at days 7, 9 and 14 only and corresponded to 3, 9 and 6 par. eq./mL of blood, respectively. The detection of this parasitaemia was consistent with the onset of the peak in rectal temperature, weight loss and major haematological changes described above. There was no detection of parasite DNA in the blood after day 14.

#### Quantitative PCR, histology and immunochemistry in tissue and organ samples

Parasite DNA was recovered from four locations in rabbit B3, displaying less than 10 par. eq. per location. The tissues and organs that yielded positive DNA amplifications were the tracheal mucosa (9 par. eq.), the vaginal mucosa (7 par. eq.), the heart apex (9 par. eq.) and the gallbladder (7 par. eq.). No positive results were obtained in skin samples and no cysts or inflammation were found in histology and immunochemistry of the positive samples.

## Discussion

No new trials studying the mechanical transmission of *B. besnoiti* by arthropod biting flies have been performed since that of Bigalke (1968). The present data demonstrated that *B. besnoiti* can be mechanically transmitted from chronically infected cows to susceptible rabbits by stable flies. This supports the prior recovery of *B. besnoiti* parasites and DNA in stable flies infected naturally (Gollnick *et al*., 2015) and experimentally (Bigalke, 1968; Liénard *et al*., 2013; Sharif *et al*., 2017) without evidence of parasite multiplication within the vectors.

Bigalke (1968) demonstrated that the persistence of *B. besnoiti* virulence in *S. calcitrans* did not exceed 1 h in a study in which trials were performed using immediate transfer between donor cows and recipient rabbits. In the present study, rabbit B3 developed clinical signs of acute besnoitiosis and seroconverted at 14 days after one exposure to potentially contaminated stable flies. Only 300 flies [a number consistent with possible numbers of flies found on cattle in summer (Todd, 1964)] were presented once to rabbit B3, whereas Bigalke (1968) estimated that 52 200–292 500 bites would be required. Estimations of the number of parasite equivalents in the mouthparts and abdominal contents of 300 *S. calcitrans* corresponded to < 50 par. eq. However, this was sufficient to trigger acute clinical signs of besnoitiosis in rabbit B3. This parasite burden was lower than in previous experimental trials in which rabbits were directly injected with 5.10^5^ to 10^7^ parasites (Shkap *et al*., 1987; Basso *et al*., 2011; Liénard *et al*., 2015). Compared with Group S, in which no clinical abnormality was observed, the clinical signs in rabbit B3 were probably attributable to transferred *B. besnoiti* only and not to adverse effects of the stable fly saliva (Swist *et al*., 2002).

The clinical signs of besnoitiosis in rabbits are inconstant following experimental infection, and vary from no or mild symptoms to an acute course of disease (Bigalke, 1960, 1967, 1968; Basson *et al*., 1970; Neuman & Nobel, 1981; Cortes *et al*., 2006; Basso *et al*., 2011). Contrary to the findings of Pols (1954) and Neuman & Nobel (1981), the present study found indications of leucopoenia and monocytopoenia, which may reflect differences in the doses used and the attenuation of virulence through 11 serial passages on rabbit of the isolate used by Pols (1954). Naturally infected cattle in the acute stage of besnoitiosis also exhibit these alterations in WBC count, including neutropoenia (Langenmayer *et al*., 2015). The peak in hyperthermia at 7 days p.e., which lasted 6 days, occurred concurrently with the detection of parasite DNA in blood, as was also reported by Basso *et al*. (2011) and Liénard *et al*. (2015). During this parasitaemia stage, regenerative anaemia with platelet consumption at 14 days p.e. probably occurred in response to the invasion of the endothelial cells by tachyzoites, leading to vascular lesions (Pols, 1960; Basson *et al*., 1970). After this acute stage, rabbit B3 recovered and appeared to be healthy at the end of the experiment.

Seroconversion was detected at 14 days p.e. by both IFAT and immunoblotting. The WB pattern was identical to that observed by Liénard *et al*. (2015) with a high antibody titre (≥ 1 : 800) determined by IFAT. None of the rabbits used here had been exposed to stable flies before the present experiments. This result may suggest either a possible adjuvant effect of stable fly saliva on immunogenicity in *B. besnoiti* or better immunological stimulation by direct inoculation of parasites in blood by vector bites in comparison with subcutaneous injection, both of which are suggested in the vector‐borne rodent malaria model (Kebaier *et al*., 2010). The possibility that different *B. besnoiti* isolates elicit heterogeneous immune responses qualitatively and quantitatively (Basson *et al*., 1970; Álvarez‐García *et al*., 2014) cannot be formally discarded.

At necropsy, the equivalent of less than 10 parasites in 500 mg of tissue of *B. besnoiti* DNA was detected in four tissues and organs from rabbit B3. Other than those in the gallbladder, these results were consistent with other findings in rabbits (Pols, 1960; Liénard *et al*., 2015), common voles *Microtus arvalis* (Rodentia: Cricetidae) (Basso *et al*., 2011) and naturally infected cattle (Frey *et al*., 2013). Histological examination failed to detect cysts. This discrepancy could be attributable to a lack of sensitivity of histological methods when the parasite burden was low (Schares *et al*., 2011; Frey *et al*., 2013). Cyst formation occurred erratically in rabbits, remaining limited even when high infective *B. besnoiti* doses were used (Pols, 1960; Bigalke, 1968; Basson *et al*., 1970; Shkap *et al*., 1987; Cortes *et al*., 2006; Basso *et al*., 2011; Liénard *et al*., 2015). Alternatively, differences in cyst forming may reflect differences between parasite isolates (Basson *et al*., 1970; Álvarez‐García *et al*., 2014).

Transmission was successful in one of three rabbits in Group B, a finding that supports the results of other trials suggesting weak vector competence in *S. calcitrans* in comparison with tabanids (Bigalke, 1968). The small numbers of recipient animals and flies may have contributed to this outcome. Transmission failure in two recipient rabbits (B1 and B2) reflected low engorgement rates on cow 1. The residual effects of pesticide treatments before hospitalization in cow 1 may explain the reluctance of *S. calcitrans* batches to take infective bloodmeals. Unfortunately, it was not possible to use the same donor in all the experiments because infected cattle must be culled quickly for welfare and clinical reasons.

## Conclusions

The success of virulent parasite transmission with 300 stable flies suggests that this common pest could contribute more effectively to the horizontal transmission of *B. besnoiti* than has been previously claimed (Bigalke, 1968). This result encourages a better consideration of the epidemiological role played by *S. calcitrans* in the local spread of cattle besnoitiosis, which should be more thoroughly investigated. Additionally, the routes involved in this mechanical transmission (e.g. inoculation by flushing of mouthparts, regurgitation) should be defined more precisely.
